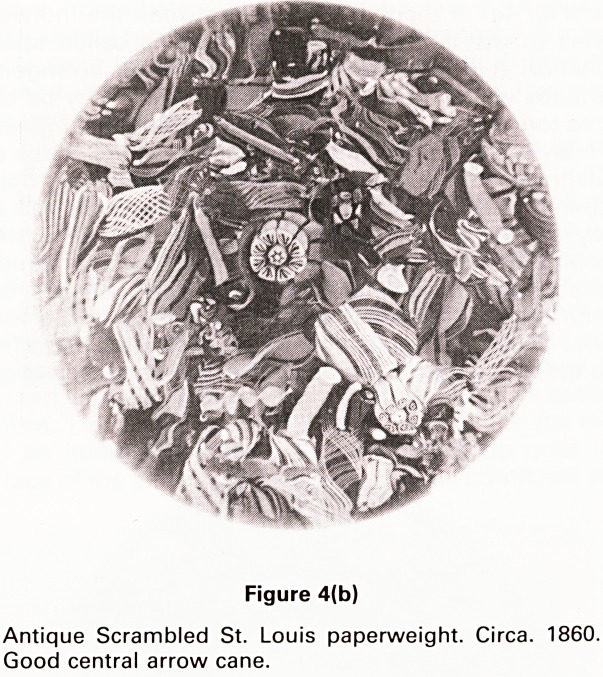# Glass Paperweights

**Published:** 1986-12

**Authors:** F. G. M. Ross

**Affiliations:** Emeritus Consultant Radiologist, United Bristol Hospitals


### Glass Paperweights

**By Dr. F. G. M. Ross, F.R.C.R., F.F.R., F.F.R.C.S.I.**
Emeritus Consultant Radiologist, United Bristol Hospitals

Glass paperweights are broadly divided into two main types. The first type is a glass sphere or plaque which encloses decorative elements such as millefiori canes, lampwork motifs, sulphide portraits and metallic motifs. 'Millefiori' in Italian literally means 'a thousand flowers' and in glasswork it refers to cross-sections of moulded glass rods which contain decorative designs. Lampwork motifs are flowers, leaves, fruit, insects etc. made of glass. The second type is the so-called 'Pinchbeck' paperweight in which the design is worked on a metal plate onto the front of which is fixed a glass dome. This type will not be considered further in this article.

### HISTORY

The history of the production of glass paperweights is obscure as is the history of glass making, the latter being traceable back to the fourteenth and fifteenth centuries B.C. Though the idea of the glass paperweight originated in Venice the main decorative element, the millefiori cane, which ensured its popularity, was Egyptian in origin. The first glass paperweights contained ceramic figures in the form of animals or portraits (Fig. 1) of well-known persons of the time, such as Napoleon. They were started in the eighteenth century in Bohemia and were developed later in France. Later still they were produced in England where Apsley Pellatt patented the process in 1819.

**Figure 1**

Modern (1970) facetted Cristal d'Albret sulphide paperweight. Portrait of HRH Prince Charles on deep blue ground. Name of subject, initials of artist (GP-Gilbert Poillerat) and date of production inscribed on base of bust.

A Venetian glassworker, Pietro Bigaglia, is generally credited with making the first paperweight containing a millefiori design which he displayed at the Austrian Industrial Fair in Vienna in 1845. Of course, he must have made them before this date in order to get them into the Fair but they were unnoticed until then. Eugene Peligot, a Professor at the Conservatoire Nationale des Arts et Métiers and a renowned glass expert was visiting the Fair as an observer for the Paris Chamber of Commerce. He was much impressed with Bigaglia's paperweights and undoubtedly brought some back to Paris. He showed them to his friends and within a year the three great well-established French glass factories (St. Louis, Baccarat and Clichy) which were not doing too well at that time, were producing paperweights of superb quality. These were much sought after and enthusiastically collected by those who set the fashions of that period. The art of making these paperweights flourished for about fifteen years after which time interest declined and their production virtually ceased, not to be revived until the early 1950's. Thus, there are two periods of Millefiori paperweight production, the so-called 'Classical Period' from about 1845 to 1860 and the 'Renaissance Period' from about 1950 onwards; these periods are also referred to as antique and modern. From its early beginnings in Venice and France, glass paperweight production spread through Europe to England about 1845, to America about 1854, to Scotland in the early 1920's and also to China in the 1930's and Japan in the 1960's. Since Paul Ysart and his family introduced the technique there in the 1920's, Scotland has been outstanding in glass paperweight production. The Strathearn Glass Company started their manufacture in 1964 (they have now ceased producing them), the Perthshire Paperweight Company started in 1968 followed by Caithness Glass Company in 1969 and Selkirk Glass Company in 1977. A more recent French factory has been producing fine sulphide paperweights under the title of Cristal d'Albret (Fig. 1) since the late 1960's.

### MANUFACTURE OF GLASS PAPERWEIGHTS

To be able to appreciate to the full glasswork, and especially paperweights, it is important to know something about the raw materials used and the methods adopted in their production. So long as it is not chipped, glass never loses any of its substance, colour or weight and this makes it an excellent medium for preserving artistic merit over the years.

The ingredients used to make the glass varies from factory to factory. The basic material is silica in the form of sand combined with an alkali, soda or lime. Up to 50% lead oxide may be added to increase its strength and improve its reflectile powers. Lead is, however, not essential and other chemicals are added in various factories to produce similar effects. The methods outlined here are those employed in the Perthshire Paperweight Company factory at Crieff in Scotland. The raw materials for the glass, chiefly white sand from the North West of Scotland, and the other carefully prepared chemicals are placed in a silica lined furnace or tank and heated by gas to above 2600°F. This process, which provides a week's supply of glass, starts on Friday afternoon and the glass, about three-quarters of a ton, is ready for use in the factory on Monday morning. When first removed from the furnace, the glass is white hot and molten. It begins to cool immediately and it then becomes viscous in which state it can be blown, rolled and shaped. It remains in this state for about 5 minutes during which time the required work on it must be completed or the glass reheated to continue the work.

A basic feature of most glass paperweights is the millefiori cane which may contain complex designs and the way these are made is fascinating in its simplicity. The worker inserts a long iron 'pontil' rod into the furnace or 'tank' and picks up a quantity or 'gather' of molten glass on its end. The gather is then rolled in a powder of coloured glass to colour it if that is required. The gather is shaped into a rounded rod by rolling it on a flat iron plate called a 'marver' after which it may be pressed vertically into an iron mould to give it an outline pattern. The pontil rod may then be returned to the tank and an additional gather of glass picked up onto the surface of the already shaped glass on the pontil rod and the whole process is then repeated, probably several times. In this way complex patterns and colours are built up within the cane. When the cane is about 6 inches long and 3 inches wide, a second pontil rod is attached by heating to its free end by another worker. The two workers then move apart and the cane is stretched between the two pontil rods, its intrinsic pattern being faithfully miniaturised as the elongation continues. Finally, when the cane is many feet long and pencil thin, it is laid on the floor, divided up into short lengths and placed in an annealing oven to cool gradually. Subsequently several of these individual canes may be bundled together to make very intricate designs, heated, coated in molten glass from the tank and then stretched to produce very complex canes. Some canes have central silhouettes in them, such as animals (Fig. 2), birds, fish or even motor cars. These canes are eventually cut across into small sections and used to form the decorative pattern of the paperweight. As well as canes, lampwork motifs are used to decorate the paperweights. In this process, small pieces of coloured glass are skilfully fashioned and then joined together with tweezers or other small tools over a blowlamp into flowers, leaves, fruit, animals, insects etc. (Fig. 3).

To make the actual paperweight, the cane sections are arranged upside down in the chosen design on an iron disc or template and heated to just below their melting point. An elongated metal collar is then placed around the edge of the template, a gather of clear glass is taken from the tank on a pontil rod, marvered into the correct shape and then lowered vertically onto the template through the collar. When the gather of glass contacts the preheated canes, the canes adhere to it. After removal from the collar, more glass is added from the tank onto the surface of the canes and used to form the dome of the paperweight. The worker then sits in his special chair, rolls the pontil rod back and forth along its arms and shapes the dome with a preformed wooden block or pad of paper held in his free hand. Using tongs, a neck is then formed between the base of the paperweight and the pontil rod, after which the neck is skilfully broken causing the paperweight to fall into a bucket of sand. The paperweight is picked up from the bucket on a spade and transferred to an annealing oven to cool for 24 hours, a a b

**Figure 2**

Antique Baccarat spaced millefiori paperweight on lace ground with date cane. (a) Top view. (b) Close up of date cane B.1847. Note inherent imperfection in form of bubbles in the glass. Many silhouette canes, some serrated, are visible including a horse, squirrel, dog, cockerel, elephant and two devils. (By courtesy of the City of Bristol Museum and Art Gallery).

**Figure 3**

Modern (1984) Perthshire Floral Spray paperweight with red flower on a white latticinio base.

process which prevents it from being damaged or deformed as cooling takes place. When cool, the paperweight is polished and it may be facetted or have a design, such as a star, cut into its base. Before facetting, it may be coated with one or more layers of contrasting coloured glass to form an overlay. From this description, it can be seen that glasswork is an almost unique art form in that the artist is unable to touch his work by hand at any time during its creation.

### CHARACTERISTICS OF THE VARIOUS TYPES OF PAPERWEIGHTS

The most uninteresting part of a paperweight is its base which may be flat or concave. Towards its centre there may be a sharp irregular area if the paperweight is antique. This is known as the pontil scar and it is the site of separation from the pontil rod. The base may also have embellishments cut into it such as a many pointed star or a grid. The profile of a paperweight is almost always dome-shaped.

The background colour or pattern on which the paperweight motif rests is called the ground. There are many well recognised types. These are clear or coloured, the colour being either translucent or opaque or Jasper which is made up of ground glass particles of two different colours. The ground may be latticinio, which consists of criss-crossing swirls of white opaque glass (Fig. 3), or lace or muslin made up of fragments of filigree twists (Fig. 2) or carpet ground which is produced by close set sections of identical millefiori canes. Overlays, already described as covering the surface of the paperweight, may be made up of one, two or three layers of opaque glass or even clear glass which may be coloured in which case it is known as 'flash'. All paperweights covered in coloured overlays must have facets or windows cut in the overlay to display the design within. The facets are on the top and around the sides. Paperweights with clear glass domes are frequently facetted too (Fig. 1). The facets increase or decrease the apparent size of the internal motif and also produce fascinating reflections.

The millefiori canes have many features. They may have finely serrated edges, the number of serrations varying from factory to factory but some were specific to individual factories. Canes which identify the factory in which the paperweight was produced and the date of manufacture were sometimes included in antique paperweights, the commonest dates being 1846, 1847 (Fig. 2), 1848 and 1849 but they are invariably included in fine modern ones. There is no recorded instance of a date cane in a paperweight made in the Clichy factory. Silhouette canes, containing simplistic likenesses of human figures, animals and plants (Fig. 2) were characteristic of single factories and have been crucial in identifying the factory of origin of antique paperweights. The Baccarat factory excelled in silhouette cane production and it featured at least seventeen different subjects. However a Rose cane was produced almost exclusively by the Clichy factory. A three-pronged arrow cane was made by both Baccarat and St. Louis (Fig. 4) but they had different characteristics by which they could be recognised.

The arrangement of the canes within paperweights follows well recognised schemes which bear specific names. The canes may be packed close together in an intricate random fashion known as closepack. When the canes are arranged in concentric circles enclosed within each other, the scheme is called concentric: if the circles of canes are separated from each other, the scheme is known as open concentric and if in contact, close concentric (Fig. 4a). If there are spaces between the individual canes in the circle, the arrangement is called spaced millefiori (Fig. 2). The canes can also be arranged in garlands which loop or intertwine or they may be clustered together in panels. The motif within a clear glass or overlay paperweight may be fashioned in the shape of a mushroom in which the canes of the tuft are grouped either closepack, close concentric or carpet ground and they are pulled out towards the base to form a stem. Around the base of such paperweights, if antique, a torsade was sometimes included. This consists of a white ring of twisted lacey cane around which a loose spiral of coloured glass was applied. When viewed from the side, this spiral slants to the right in paperweights

**Figure 4(a)**

Antique Baccarat close concentric paperweight circa. 1860. (i) Top view (ii) Close up of typical arrow canes of Baccarat factory Note inherent imperfection of tilted canes in inner ring.

**Figure 4(b)**

Antique Scrambled St. Louis paperweight. Circa. 1860. Good central arrow cane.

made by St. Louis and to the left in Baccarat paperweights. The commonest type of antique paperweight was the scrambled or 'end-of-the-day' weight; these were made up, frequently by apprentices, of bits and pieces of all the ingredients of a paperweight producing an overall random effect (Fig. 4b).

Representational paperweights are so called because their motifs represent flowers (Fig. 3), fruit and various forms of animal and insect life. They are produced by lampwork and they frequently include millefiori canes but, if antique, never date canes. They may take the form of a colourful pansy, primrose, clematis, etc., or fruit usually mixed, such as pears and cherries with green leaves or insects like a butterfly or even a snake. Recently motifs consisting chiefly of bubbles in abstract design and paperweights made of glass coated in iridescent colours have been introduced by newer factories in Scotland and England.

### DISCUSSION

In an article of this length it is quite impossible to describe all the features of glass paperweights and much has had to be left out. In addition it is important to handle them and look at them from differing angles and in various lighting conditions so that the beauty of the colouring, the reflections and magnifications can be highlighted. When one appreciates how they are made and designed in a medium that cannot be directly touched by the artist, their fascination can be easily understood. Added to this is the fact that no two paperweights can be identical and this has collector appeal. Why they have been so avidly collected since about 1845 is not easily explainable because in that time there has not been any practical need for an expensive piece of glass to keep papers in their place. Their undoubted appeal must rest on their colourfulness and beauty which do not fade with time.

### ACKNOWLEDGEMENTS

I am most grateful to Mr. Stuart Drysdale, Managing Director of the Perthshire Paperweight Company, Crieff, Scotland, for allowing me to witness, photograph and describe the many processes in the production of glass paperweights at his factory, to the Curator of the City of Bristol Museum and Art Gallery for Figure 2, to Mr. J. Hancock for the reproductions and to Miss Carol Phillips for typing the manuscript.

## Figures and Tables

**Figure 1 f1:**
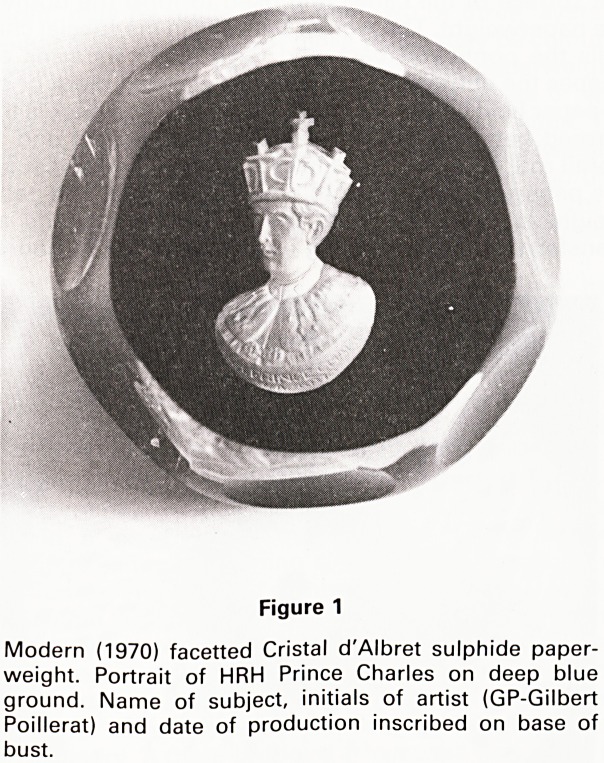


**Figure 2 f2:**
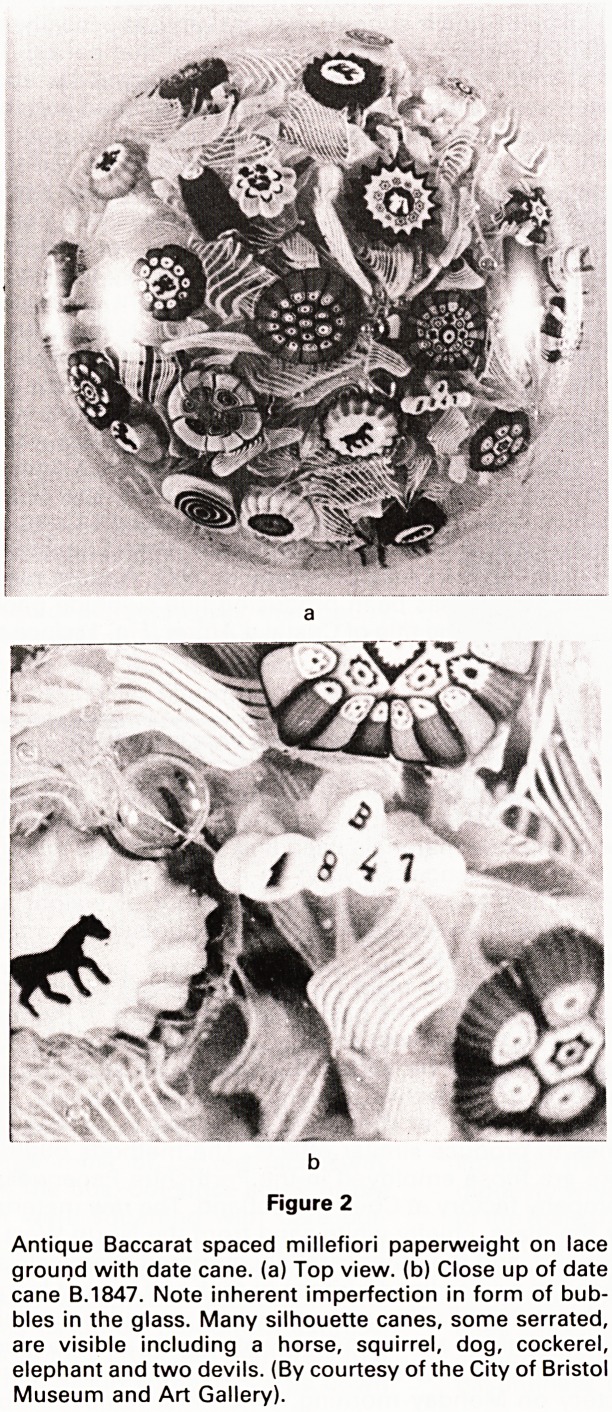


**Figure 3 f3:**
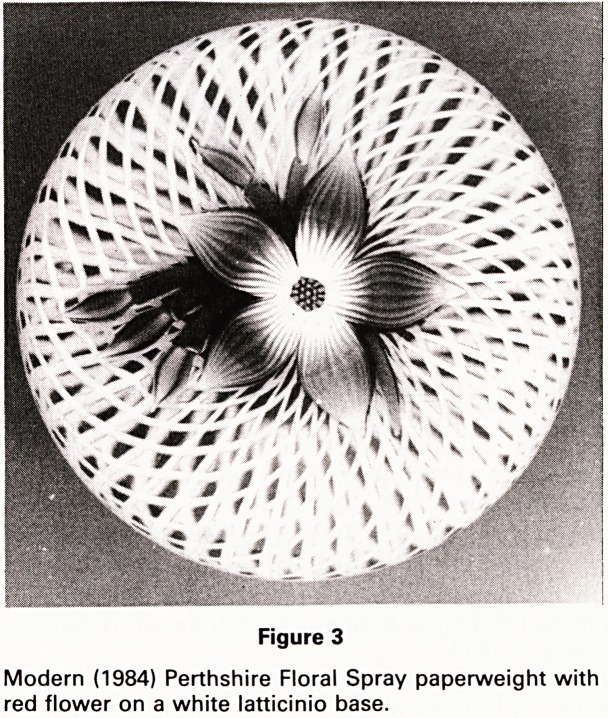


**Figure 4(a) f4:**
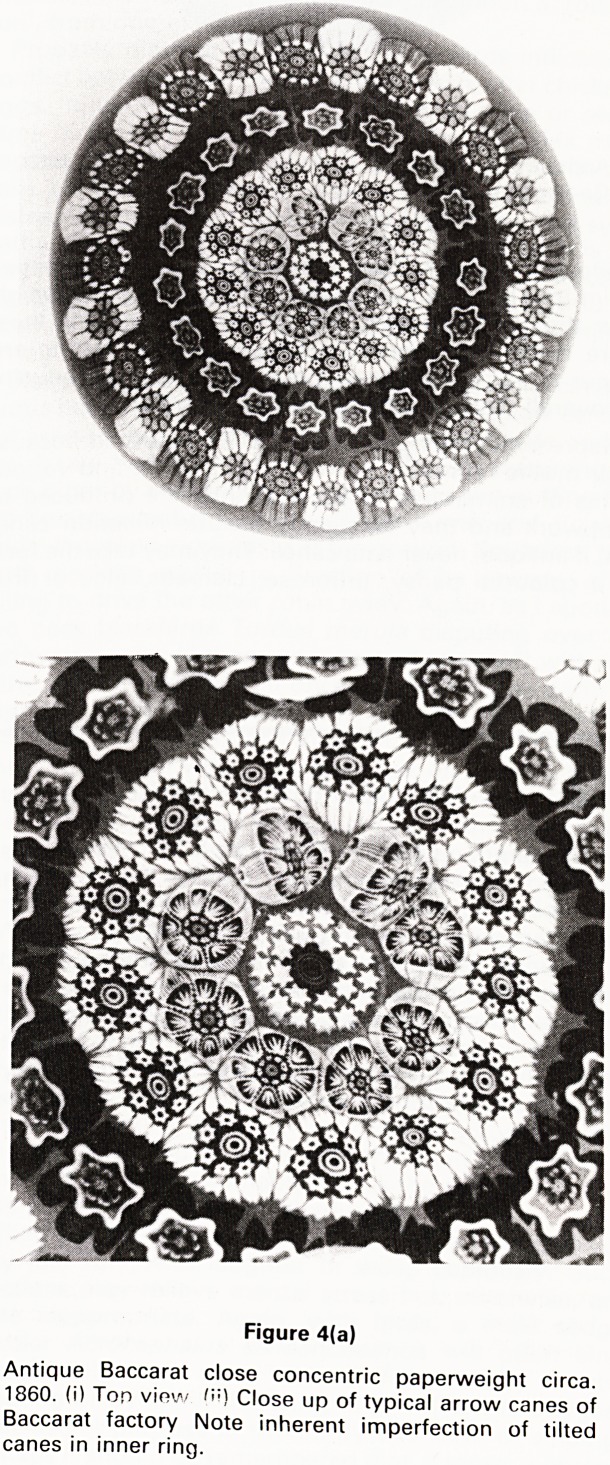


**Figure 4(b) f5:**